# Associations between food allergy, country of residence, and healthcare access

**DOI:** 10.1186/s13223-022-00745-4

**Published:** 2022-12-06

**Authors:** Kaitlyn A. Merrill, Elissa M. Abrams, Sara V. Good, Ruchi S. Gupta, Carina Venter, Tara Lynn M. Frykas, Michael A. Golding, Jennifer L. P. Protudjer

**Affiliations:** 1 Department of Pediatrics and Child Health, Rady Faculty of Health Sciences, Max Rady College of Medicine, University of Manitoba, Winnipeg, MB Canada; 2 Children’s Hospital Research Institute of Manitoba, Winnipeg, MB Canada; 3 Division of Allergy and Immunology, Department of Pediatrics, Faculty of Medicine, University of British Columbia, Vancouver, BC Canada; 4 Department of Biology, Faculty of Science, University of Winnipeg, Winnipeg, MB Canada; 5 Department of Biological Sciences, Faculty of Science, University of Manitoba, Winnipeg, MB Canada; 6 Feinberg School of Medicine, Northwestern University, Chicago, IL USA; 7 Pediatric Allergy and Immunology, University of Colorado, Denver, CO USA; 8 Department of Food and Human Nutritional Sciences, Faculty of Agricultural and Food Sciences, University of Manitoba, Winnipeg, MB Canada; 9 George and Fay Yee Centre for Healthcare Innovation, Winnipeg, MB Canada; 10 Centre for Allergy Research, Karolinska Institutet, Stockholm, Sweden

**Keywords:** Food allergy, Oral food challenge, Canadian healthcare, United States healthcare, Healthcare access

## Abstract

**Background:**

To date, little consideration has been given to access to allergy-related care, despite the fact that food allergy affects a considerable proportion of children. As such, the current study aimed to describe access to food allergy-related services in Canada and the United States (US).

**Methods:**

Participants were recruited via social media from March-July 2021 and were asked to complete an online survey focused on food allergy-related medical care. Participants were Canadian and US residents who live with a child < 18 years old, with ≥ 1 food allergy. A series of logistic regressions were used to assess the associations between country of residence and type of allergy testing utilized during diagnosis.

**Results:**

Fifty-nine participants were included in the analysis (Canadian: 32/59; 54.2%; US residents: 27/59; 45.8%). Relative to Canadian participants, US respondents were less likely to be diagnosed using an oral food challenge (OFC; OR 0.16; 95% CI 0.04; 0.75: p < 0.05). Compared to children diagnosed by age 2 years, those diagnosed at age 3 years and older were less likely to have been diagnosed using an OFC (OR 0.12; 95% CI 0.01; 1.01; p = 0.05).

**Conclusions:**

Access to food allergy-related services, varies between Canada and the US. We speculate that this variation may reflect differences in clinical practice and types of insurance coverage. Findings also underscore the need for more research centered on food allergy-related health care, specifically diagnostic testing, among larger and more diverse samples.

Despite being neighbouring countries, the healthcare systems of Canada and the United States (US) are vastly different. Under the Canada Health Act, Canadian citizens are able to access health services without incurring direct expenses, including allergy-related care and allergy testing [1]. Although this is a federal act, provincial and territorial governments are directly responsible for service provision, meaning that all Canadian citizens have provincial or territorial health insurance [2]. It is important to note that, despite universal healthcare, provision of healthcare can vary province-to-province [3]. In contrast to Canada’s universal healthcare model, US citizens either must pay for private insurance, obtain supplemented private insurance through work benefits, use government supports (Medicaid/Medicare) or pay for medical services, including food allergy care, out-of-pocket [4]. Not all US residents are able to apply to receive Medicaid (designed to support low income citizens), leaving many (~ 16% of US population) uninsured in times of medical crisis, which is a significant cause of bankruptcy in the US [2, 4]. Given that little is known about the differences in the diagnostic process between the two countries, and that correct and timely diagnosis of allergies are of great importance, this topic is of interest.

In combination with a clinical history, three common tests are used to diagnose or rule out food allergy. These include: skin prick tests (SPT), serum-specific immunoglobulin E (sIgE) antibody tests and oral food challenge (OFC) [5]. SPTs and sIgE are widely available, however, show high rates of false positive results due to high sensitivity and low specificity which can lead to unnecessary food avoidances, and there is evidence to suggest that many children who show sensitization to a food using these tests are able to tolerate the food during OFC [6, 7]. OFCs are considered to be the “gold standard for allergy diagnosis”, but an OFC is the most costly and time-consuming diagnostic test [6]. The limited availability of OFCs, over-reporting of food allergy, and the widespread use of food avoidance due to perceived allergic reactions, suggests that there are a significant number of children living with unnecessary food avoidance, undue anxiety that accompanies living with a food allergy and decreased health-related quality of life [8–14]. We hypothesise between Canada and the United States, the type of allergy testing used to diagnose pediatric food allergy will differ significantly.

The study used an online, anonymous survey disseminated on social media between March and early July 2021. Analyses include descriptive statistics (n/N, %, mean ± standard deviation [SD]) of various food allergy prevalence within the sample. We then used chi-square and Fisher exact tests, as well as logistic regression (reported as odds ratio [OR] and 95 per cent confidence intervals [95% CI]) to determine associations between country of residence (US vs. Canada), food allergy testing performed during diagnosis and medical insurance (no vs yes, by type). Data were analyzed using Stata® (Version 17 College Station, TX). Logistic regression models were developed using the variables of interest described in the hypothesis, namely the type of allergic test performed at diagnosis and the country of residence. The study received approval from the University of Manitoba Research Ethics Board, ethics file number HS24604 (H2021:034).

The survey yielded 59 participants, 32 (54%) reported being from Canada (> 90% from Manitoba) and 27 (45.8%) reported being from the US (evenly spread country-wide). With consideration to the use of OFCs as a diagnostic test, compared to participants in Canada, those in the US had lower odds of being tested via OFC in the unadjusted (OR 0.33; 95% CI 0.10; 1.07; p < 0.10), adjusted (e.g., adjusted for: age at diagnosis [aOR 0.21; 95% CI 0.06; 0.76; p < 0.05]) and fully adjusted models (adjusted for: age at diagnosis and annual household income [aOR 0.16; 95% CI 0.04;0.75: p < 0.05]; Fig. 1 and Table 1).

**Fig. 1:**
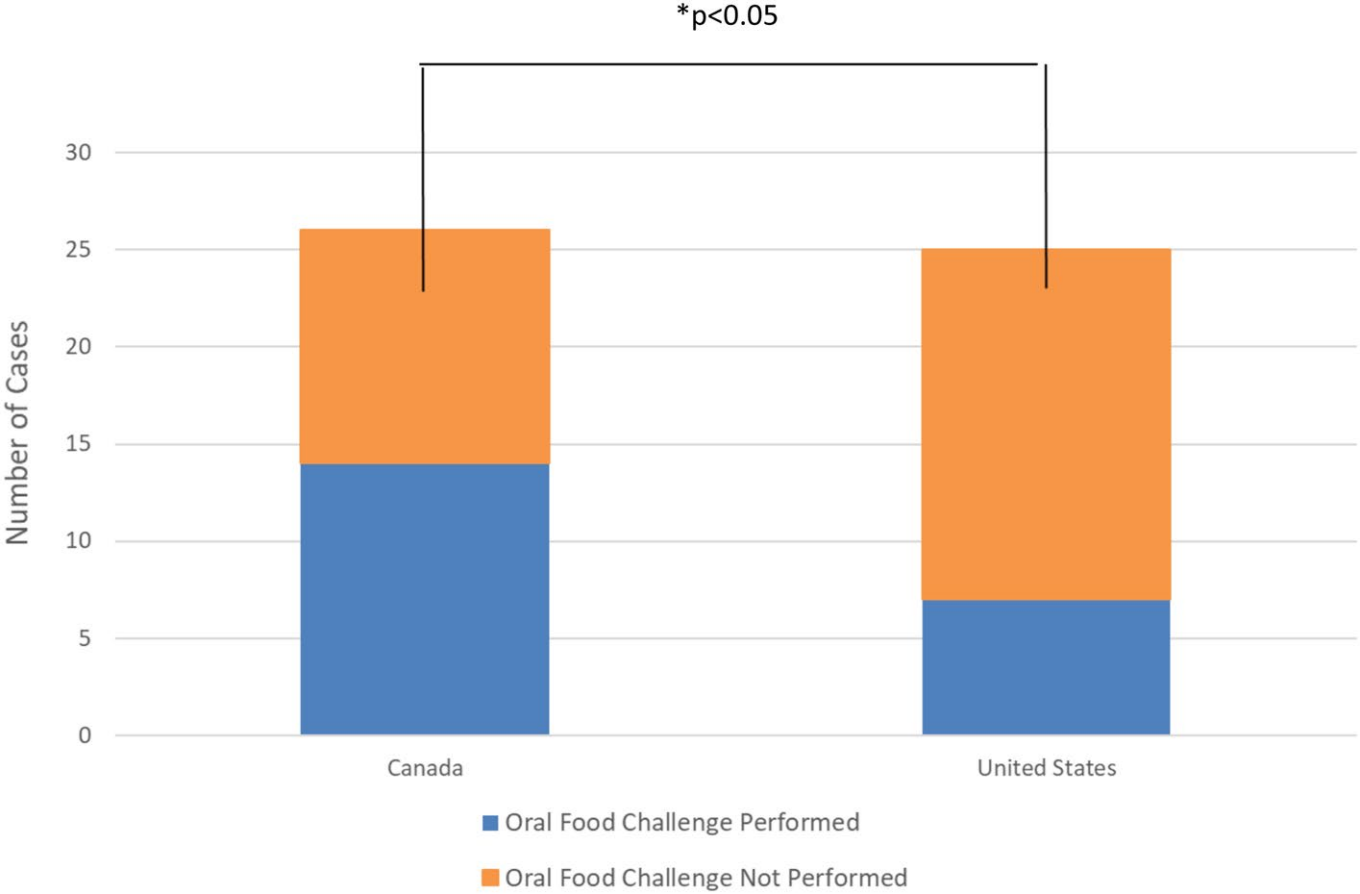
Association between number of oral food challenge diagnostic tests provided in Canada vs. the US

**Table 1:** Logistic regression analysis of the association between oral food challenges (OFC) and country of residence (N = 53)

n	Unadjusted		Model 1*		Model 2^†^	
	OR	95% CI	OR	95% CI	OR	95% CI
Canada	1.00		1.00		1.00	
US	0.33	0.10; 1.07	0.21^‡^	0.06; 0.76	0.16^‡^	0.04; 0.75

^*^Adjusted for age at diagnosis

^†^Adjusted for age at diagnosis and annual household income

^‡^p < 0.05

Age at diagnosis was also associated with OFC. Compared to children aged ≤ 2 years, children aged ≥ 3 years had a significantly lower odds of having an OFC (OR 0.05; 95% CI 0.00; 0.59; p < 0.02); Table 2).

**Table 2:** Logistic regression analysis of the association between age at diagnosis and oral food challenges (OFC) (N = 51)

	n	Unadjusted		Model 1*		Model 2^†^	
		OR	95% CI	OR	95% CI	OR	95% CI
≤ 2 yo	46	1.00		1.00		1.00	
≥ 3 yo	11	0.17	0.10; 1.01	0.07^‡^	0.01; 0.65	0.05^‡^	0.00; 0.59

^*^Adjusted for country of residence

^†^Adjusted country of residence and annual household income

^‡^p < 0.05

In this online, anonymous survey-based study of parents of children with food allergy, we found that Canadians were significantly more likely (70%) than US residents to obtain an OFC. This finding may be due to the differences in cost of care incurred by US residents if their insurance does not fully or partially cover the costs of an OFC. A US study determined that there are numerous practical barriers that US allergists report for not performing OFCs as often as they should, including a lack of time, space and staffing [15], and the possible risk of litigation. These barriers may also provide reasoning for the discrepancies in the rate of OFC provided between Canada and US. This study is not without limitations, the most notable of which is the small sample size. In addition, majority of Canadian participants reside in Manitoba, and as such, the findings may not be generalizable to other provinces.

In conclusion, this study provides evidence that the differences between Canadian and US healthcare systems impact the type of care received by patients. Findings also highlight the need for additional research centered on food allergy-related health care and types of allergy testing utilized in larger, more diverse and more geographically spread samples.

## Data Availability

Anonymous data are available from the corresponding author upon reasonable, written request.

## Abbreviations

aOR: Adjusted odds ratio
EAI: Epinephrine auto-injector
IgE: Immunoglobulin E
OFC: Oral food challenge
OR: Odds ratio
SD: Standard deviation
SPT: Skin prick test
US: United States
